# Influence of Porosity of Sulfide-Based Artificial Solid Electrolyte Interphases on Their Performance with Liquid and Solid Electrolytes in Li and Na Metal Batteries

**DOI:** 10.1021/acsami.1c23923

**Published:** 2022-03-31

**Authors:** Kyungmi Lim, Bernhard Fenk, Kathrin Küster, Tolga Acartürk, Jürgen Weiss, Ulrich Starke, Jelena Popovic, Joachim Maier

**Affiliations:** Max Planck Institute for Solid State Research, 70569 Stuttgart, Germany

**Keywords:** porosity, solid-state batteries, sulfide-base electrolytes, solid-electrolyte interphase, lithium, sodium

## Abstract

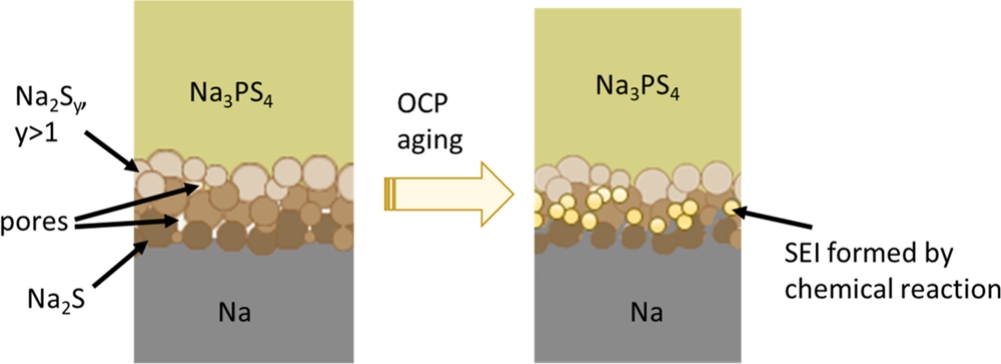

Realization of all-solid-state batteries combined with metallic Li/Na is still hindered due to the unstable interface between the alkali metal and solid electrolytes, especially for highly promising thiophosphate materials. Artificial and uniform solid-electrolyte interphases (SEIs), serving as thin ion-conducting films, have been considered as a strategy to overcome the issues of such reactive interfaces. Here, we synthesized sulfide-based artificial SEIs (Li_*x*_S_*y*_ and Na_*x*_S_*y*_) on Li and Na by solid/gas reaction between the alkali metal and S vapor. The synthesized films are carefully characterized with various chemical/electrochemical techniques. We show that these artificial SEIs are not beneficial from an application point of view since they either contribute to additional resistances (Li) or do not prevent reactions at the alkali metal/electrolyte interface (Na). We show that Na_*x*_S_*y*_ is more porous than Li_*x*_S_*y*_, supported by (i) its rough morphology observed by focused ion beam-scanning electron microscopy, (ii) the rapid decrease of *R*_interface_ (interfacial resistance) in Na_*x*_S_*y*_-covered-Na symmetric cells with liquid electrolyte upon aging under open-circuit potential, and (iii) the increase of *R*_interface_ in Na_*x*_S_*y*_-covered-Na solid-state symmetric cells with Na_3_PS_4_ electrolyte. The porous SEI allows the penetration of liquid electrolyte or alkali metal creep through its pores, resulting in a continuous chemical reaction. Hence, porosity of SEIs in general should be carefully taken into account in the application of batteries containing both liquid electrolyte and solid electrolyte.

## 1 Introduction

Owing to the climate change crisis and energy shortages, efficient energy storage has become an aspirational goal of mankind.^1^ The most demanding aspect of “efficiency” in energy storage systems, in particular in mobile applications, would most likely be the energy density, which describes the amount of energy stored in a specific volume/weight. Solid-state batteries (SSBs), combined with alkali metals, have been attracting attention as a new energy storage system that can meet the needs of the times since their volumetric/gravimetric energy density is higher than in conventional Li batteries.^2,3^ However, interfacial issues hinder the application of SSBs, especially for sulfide-based solid electrolytes known to have high ionic conductivities in the order of ∼10^–3^ S cm^–1^.^4,5^ Interfacial issues include high resistances stemming from the imperfect electrode/electrolyte contact, a high activation energy barrier for charge transfer at grain boundaries (or at phase boundaries), and a continuous growth of the solid-electrolyte interphases (SEIs) on reactive alkali metals linked with the formation of mixed-conducting SEIs.^4,6−8^ Additionally, the chemically inhomogeneous interface between alkali metal and the solid electrolyte induces uneven ion transport, leading to the preferential stripping/plating at certain positions and, consequently, the formation of dendrites.^9^ Introducing artificial SEIs (*i.e.*, SEIs which are formed *ex situ* prior to the electrochemical cell assembly), if carefully chosen, can be a strategy to overcome these issues. Chemically stable and homogeneous artificial SEIs have already been proposed for Li/Na metal batteries when liquid electrolytes are used, demonstrating stable galvanostatic stripping/plating cycleability.^10−17^ Meanwhile, much fewer reports focus on the role of artificial SEIs on the Li/Na metal with reactive solid electrolytes,^18−22^ and systematic comparative studies of Li and Na are scarce. The underlying reason might be the complexity of the SSB system, where many chemo-mechanical parameters affecting the cell performance are intertwined with one another. For Li/Na metal batteries where liquid electrolytes are employed, mechanical problems are less relevant compared to SSBs since the liquid electrolyte penetrates through the pores and touches all surfaces of the electrode particles evenly, maintaining good contact.^2^ On the contrary, a number of issues in SSBs originate not only from chemical but also from mechanical instabilities of the interfaces. These two effects may also be correlated to each other.^23^ For instance, external pressure applied to the cell improves the contact between the electrode and electrode/electrolyte particles but simultaneously induces Li or Na metal creep, leading to the Li/Na penetration through voids and further chemical reactions.^24^ If the molar volume of the reactant produced by the chemical reaction is different from the already existing ones, the contact may be lost again during volume contraction and/or expansion.^25,26^ Therefore, introducing another interface such as an artificial SEI in SSBs should be carefully considered as it might result in even more complex chemo-mechanical processes.

In this work, we introduce sulfide-based artificial SEIs on Li/Na metals and systematically compare their roles in battery cells with liquid and solid electrolytes. Li_2_S and Na_2_S both have antifluorite structures and are known to be chemically stable against Li and Na, respectively.^27−29^ They have been shown to be predominantly ionic conductors with ionic conductivities of ∼10^–11^ S cm^–1^ (Li_2_S) and ∼10^–10^ S cm^–1^ (Na_2_S) at room temperature,^30,31^ but note that the ionic conductivity values are not invariant under transformation. They may not only be influenced by the detailed composition, but have also been reported to increase (to up to ∼10^–8^ S cm^–1^) upon nano-sizing^32,33^ owing to the changes in the bulk defect chemistry or to space charge effects. For the above reasons, Li_2_S and Na_2_S are expected to be able to prevent further chemical reactions between Li/Na and electrolytes. More precisely, although the electronic conductivity directly at the contact cannot be excluded, the chemical transport of Li(Na) is expected to be sluggish if the tunneling distance is exceeded. In addition, Li_2_S and Na_2_S can be easily synthesized *via* Li(Na) exposure to sulfur vapor,^34,35^ which has an advantage over the solvent-mediated method^36^ since only pure S is included as a reactant, avoiding the risk of unwanted side reactions with Li/Na. Also, due to the fact that Li_2_S and Na_2_S share similarities in the crystallographic structure and ion conduction behavior, a comparative study of their roles in battery operation may allow exclusion of the chemical factors stemming from the SEI and may provide insights into the morphological/mechanical difference of Li and Na. The sulfide-based layers grown on Li/Na are systematically investigated with focused ion beam-scanning electron microscopy (FIB-SEM), X-ray diffraction (XRD), X-ray photoelectron spectroscopy (XPS), and time-of-flight secondary ion mass spectroscopy (ToF-SIMS). Finally, their role as artificial SEIs in symmetric Li(Na) metal cells is examined with electrochemical impedance spectroscopy (EIS) and galvanostatic stripping/plating. Growth of the sulfide-based artificial SEIs and electrical conduction behavior in Li and Na system are systematically compared.

## 2 Experimental Section

### 2.1 Synthesis of Li_*x*_S_*y*_(Na_*x*_S_*y*_) on Li(Na)

**Figure 1:**
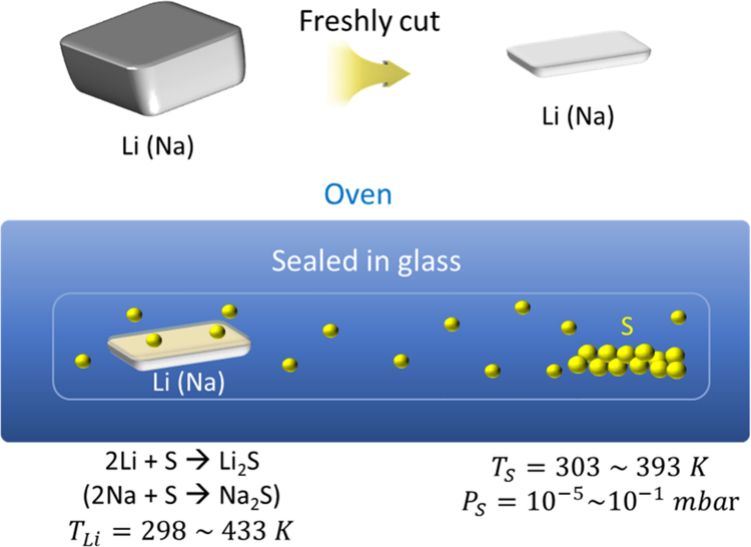
Schematic diagram of the synthetic method used for preparation of artificial sulfide-based SEIs. *T*_S_, temperature of sulfur; *P*_S_, vapor pressure of sulfur; and *T*_Li_, temperature of Li.

The Li rod (99.9%, trace metals basis) and Na cubes stored in a mineral oil (99.9%, trace metals basis) were purchased from Sigma-Aldrich and were stored in a glovebox under an Ar atmosphere. S powder (99.998%, trace metals basis, Sigma-Aldrich) was dried in a vacuum oven at 100 °C overnight before use. Li and Na metals were wiped with a tissue paper to remove the residual oil and freshly cut with stainless-steel blades. Subsequently, they were sandwiched between two Celgard separators and roll-pressed to approximately the same thickness (0.15 mm). The Celgard separator was used during pressing since Li and Na adhere strongly to the stainless-steel pressing plate. After the Li(Na) pressing procedure, discs with a diameter of 10 mm were cut and attached to stainless steel discs with a diameter of 18 mm. The stainless-steel discs act as a supporting substrate since the alkali metals easily lose their form. The so-prepared electrodes were placed in homemade glass ampoules with a length of 600 mm together with the S powder. The glass ampoule was placed in an oven and heated at different temperatures (in the temperature range from 298 to 433 K). All processes were performed in a glovebox (both O_2_ and H_2_O levels were below 0.1 ppm). A schematic diagram of the Li_*x*_S_*y*_(Na_*x*_S_*y*_) synthesis is shown in Figure 1.

### 2.2 Characterization of Li_*x*_S_*y*_(Na_*x*_S_*y*_) Formed on Li(Na)

For FIB-SEM analysis, samples were transferred from the Ar-filled glovebox to the SEM chamber with a homemade airtight transfer tool. Cross-section images were measured by using a Zeiss Crossbeam scanning electron micoscope with a built-in focused ion beam (FIB). FIB cutting was performed using a Ga^+^ beam (accelerating voltage: 30 kV) with a current ranging from 50 pA to 2 nA, depending on the sample and its reactivity.

XPS of Li samples was performed on a Kratos Axis Ultra system with a monochromatic Al Kα X-ray source. High-resolution data were acquired with a pass energy of 20 eV. Ar^+^ sputtering was performed using a scanned Minibeam III sputter gun (Kratos) with a beam energy of 4 kV and an emission current of 20 mA. XPS data were analyzed with CasaXPS software (version 2.3.23PR1.0 by Casa Software Ltd.). For accurate phase identification, the peak shift due to charging was corrected based on the binding energy of Li_2_S,^37^ and the phase identification was confirmed again with the binding energy separation between O 1s and Li 1s in Li_2_O as discussed in the literature.^38^ For fitting of the S 2p peaks, the binding energy difference between the spin–orbit split components (S 2p_3/2_ and S 2p_1/2_) and their area ratio were constrained to 1.2 eV and 2:1, respectively.^39^ Samples were transferred to the XPS chamber under an Ar atmosphere in an airtight transfer tool.

ToF-SIMS measurements were carried out using a ToF-SIMS V instrument (ION-TOF) version NCS. Dual beam depth profiles were acquired using a cyclic sputter-probe series. The sputter gun and the analysis gun were operated in the noninterlaced mode with an additional flood gun in order to avoid sample charging. For sputtering, a Cs^+^ source was employed with 2 keV ion energy and 132 nA current on a 500 × 500 μm^2^ crater area. For probing, monoatomic Bi_1_^+^ ions were used, accelerated by 30 keV, with a current of 1.7 pA, on a 100 × 100 μm^2^ analysis area. Ions were acquired with negative polarity. Data analysis was carried out using the software Surfacelab 7.1. The ion intensities were normalized to the total ion count for each sputter time data point. Samples were transferred under an Ar atmosphere to the chamber using a commercial (ION-TOF) airtight transfer vessel.

XRD was measured using an X-ray diffractometer from PANalytical GmbH (Empyrean Series 2) with a Cu Kα radiation energy of 40 kV and a current of 40 mA. The samples were mounted in a domed airtight sample holder from Anton Paar. For phase determination, HighScore Plus software, version 3.0e, was used.

### 2.3 Synthesis of Li_3_PS_4_ and Na_3_PS_4_

β-Li_3_PS_4_ was synthesized *via* a solvent-mediated synthesis as reported in the literature.^40−45^ Li_2_S (99.9%, Sigma-Aldrich) and P_2_S_5_ (98+%, Acros Organics) were mixed in a 3:1 molar ratio and poured to tetrahydrofuran (Fischer Scientific, anhydrous, synthesis grade) with a volume ratio of 1:20. The mixture was stirred for 24 h at room temperature under an Ar atmosphere. Subsequently, the mixture was centrifuged at 9000 rpm for 10 min and dried under vacuum at 140 °C for 24 h.

Na_3_PS_4_ was synthesized *via* mechanical ball milling.^46,47^ Na_2_S (nonhydrate, extra pure, Fischer Scientific) and P_2_S_5_ (anhydrous, synthesis grade, Fischer Scientific) were mixed in a 3:1 molar ratio and put in a ZrO_2_ jar with five ZrO_2_ balls. The ball-to-sample weight ratio was 7:1. The powder mixture was milled using a planetary mill apparatus (Fritsch, planetary mill apparatus Pulverisette 5) at 250 rpm for 48 h. The samples were neither heat-treated nor exposed to air during the synthesis and handling procedure.

### 2.4 Cell Assembly and Electrochemical Measurements

CR2032-type coin cells made of stainless steel were assembled with two Li(Na) symmetric electrodes and liquid or solid electrolytes. Liquid electrolytes were prepared by dissolving 1 M LiCF_3_SO_3_ (98%, Sigma-Aldrich) and 1 M NaCF_3_SO_3_ (99.5%, Solvionic) into triethylene glycol dimethyl ether (triglyme, 99%, Alfa Aesar). The H_2_O content in the liquid electrolyte was controlled to be below 1 ppm (using the Karl Fischer titration technique). For the cells with liquid electrolytes, two symmetric electrodes were separated by a 20 μm-thick Celgard separator (H2013) soaked with 20 μL of the liquid electrolyte. Here, a spring was included in the cell for achieving better contact between the different components. For the solid-state cells, the synthesized solid electrolyte powders (Li_3_PS_4_ and Na_3_PS_4_) were pressed uniaxially (380 MPa, 10 mm diameter, between 70 and 80% density) in the glovebox and sandwiched between the two Li(Na) electrodes in a coin cell. In solid-state cells, a spring was not used due to the limited cell thickness. In order to determine the ionic conductivity of the solid electrolytes, the pellets were produced in the same way as previously described but were sputtered with Ru (400 nm thickness) on both flat sides in the glovebox.

EIS was performed starting from 10^6^ to 0.1 or 1 Hz, depending on the specific cells. EIS was conducted in the potentiostatic mode, with an amplitude of 0.1 V, using Solartron 1260 and Novocontrol Alpha-A devices. Temperature-dependent EIS measurements were performed in the temperature range from 80 to 0 °C for 1 h for each temperature using an external thermostat (Lauda RC6CP). Impedance spectra were analyzed with ZView software (Scribner Associates, version 3.5c).

## 3 Results and Discussion

Figure 1 illustrates the synthesis route for obtaining sulfide-based artificial SEIs on Li/Na. Since the ampoule containing Li/Na and S is long enough, the temperature of Li/Na [*T*_Li(Na)_] and the temperature of S (*T*_S_) could be regulated separately, allowing subtle control of reaction kinetics by fixing the vapor pressure of S and the reaction constant. The temperature on the Li/Na side of the ampoule was kept at the same or higher value than the temperature on the S side in order to avoid the condensation of S vapor. The expected solid–vapor chemical reaction is as follows^48,49^
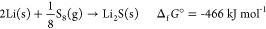

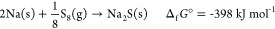
In contrast to the fact that Li_2_S is the only thermodynamically stable phase in the Li–S system,^50^ the possibility of formation of sodium polysulfides such as Na_2_S_2_, Na_2_S_4_, and Na_2_S_5_ cannot be excluded in the Na case.^51^

**Figure 2:**
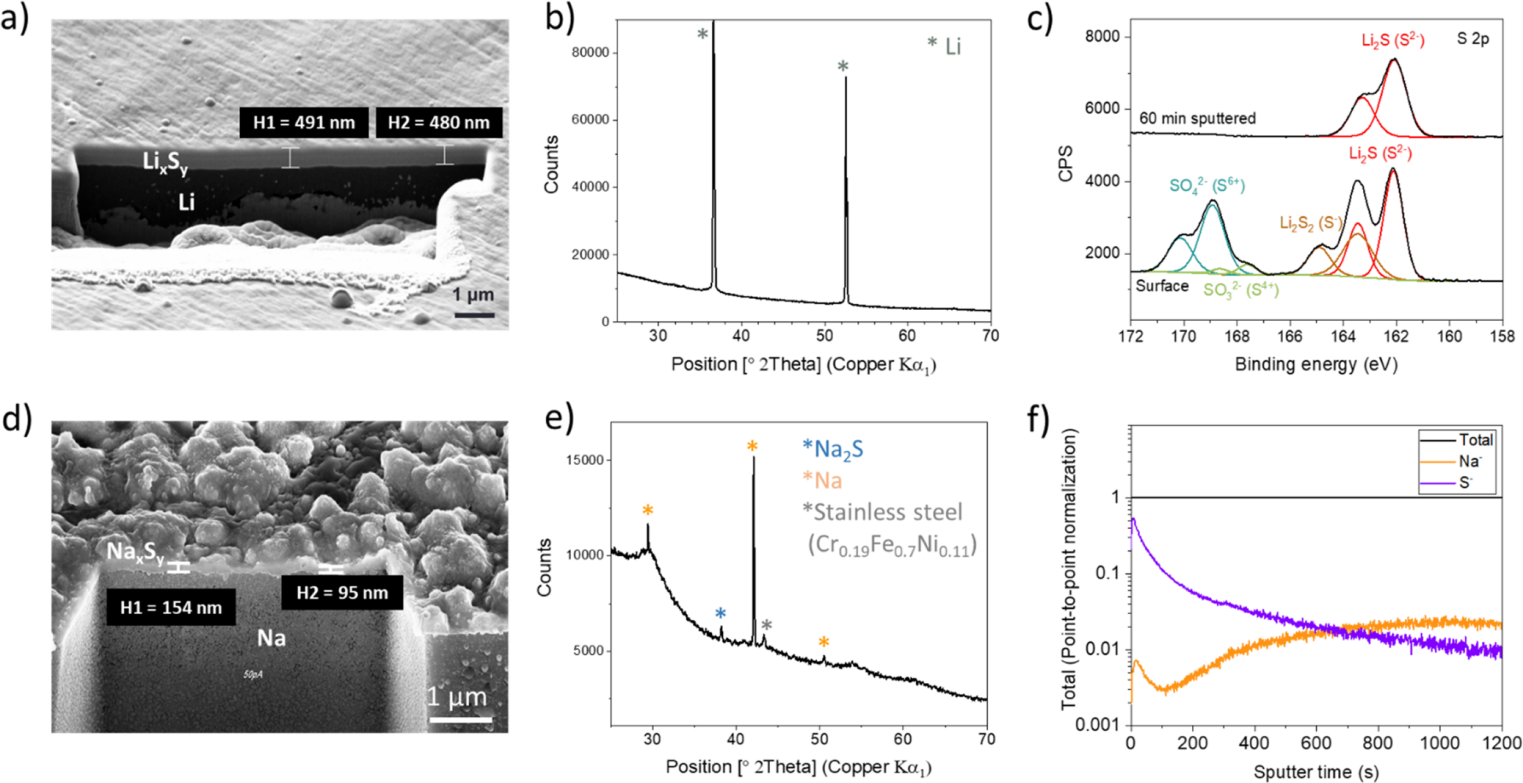
Characterization of sulfide-based artificial SEIs on Li (top row) and Na (bottom row). (a,d) Cross-section images taken by FIB-SEM. (b,e) XRD patterns. (c) XPS spectra (black line) collected on the surface of Li_*x*_S_*y*_ and upon Ar^+^ etching (top). The spectra were shifted for better visibility. The red, brown, green, and blue lines correspond to the fitting of the total spectra. (f) ToF-SIMS depth profile of Na_*x*_S_*y*_. L_i*x*_S_*y*_ was synthesized at *T*_Li_ = 140 °C and *T*_S_ = 25 °C for 2 h, and Na_*x*_S_*y*_ was synthesized at *T*_Na_ = *T*_S_ = 80 °C for 2 h.

Figure 2 shows the data collected from a variety of materials’ characterization techniques employed on the synthesized artificial SEI films. Cross-section images measured by FIB-SEM in Figure 2a,d reveal the morphology of the Li_*x*_S_*y*_ and Na_*x*_S_*y*_ films. In both cases, the thickness and morphology of the films depend on the synthesis temperature (see Figures S1 and S2 in the Supporting Information), and the overall observation is that the film thickness increases with increasing *T*_Li(Na)_ and *T*_S_. In addition, Na_*x*_S_*y*_ appears to have a rougher surface than Li_*x*_S_*y*_, which might indicate a higher porosity at the nanoscale. This may be attributable to the inherently partially porous native film on Na,^52^ which may have led to the porous film even after the chemical reaction. Another contribution could be the molar volume difference between the reactant (Na) and products (Na_2_S, Na_2_S_2_, Na_2_S_4_, and Na_2_S_5_), described by the Pilling–Bedworth ratio (*R*_PB_, relative molar volume increase during sulfidation). For Na_2_S, *R*_PB_ is less than 1, suggesting that a porous film is favored, while sodium polysulfides with *R*_PB_ values greater than 1 induce compressive stress, favoring crack formation in the SEI (see Table S1, Supporting Information). Additionally, energy-dispersive X-ray spectra confirm the existence of S (Figure S3, Supporting Information) in the synthesized layer. The artificial sulfide-based SEI on Na is a crystalline film (Figure 2e), while the one on Li might be amorphous (Figure 2b). However, it should not be neglected that the intensity of the diffracted X-rays is a function of the atomic number. Since Na has a higher atomic number than Li, the probability of X-ray scattering in Na atoms is also higher than in Li, resulting in an increased form factor and scattering intensity. Finally, depth profiling of the chemical composition of the film was performed by XPS and ToF-SIMS. XPS data (Figure 2c) show that the surface of the film on Li is composed of a variety of sulfur-related compounds such as sulfate (SO_4_^2–^), sulfite (SO_3_^2–^), polysulfide (Li_2_S_*y*_, *y* > 1), and sulfide (Li_2_S), while the expected Li_2_S phase is present in the bulk (for more information about phase identification by XPS, see Experimental Section2.2; Table S2 in the Supporting Information provides the peak positions and the reference of XPS analysis in each phase). A similar chemical complexity was reported for sputter-deposited lithium sulfide films in the literature where the surface was covered with lithium polysulfides and the bulk was composed of Li_2_S.^53^ We could not perform XPS measurements on Na due to its relatively high vapor pressure (∼10^–8^ mbar^54^), which might lead to contaminations of the vacuum chamber. Therefore, ToF-SIMS was applied to characterize the Na_*x*_S_*y*_ film on Na (Figure 2f). Note that ToF-SIMS analysis for Na_*x*_S_*y*_ on Na is qualitative, not quantitative, since the sample is rough, inhomogeneous, and reactive. As the sputtering in ToF-SIMS progresses, first, the concentration of Na decreases and then gradually increases, while the concentration of S decreases. The results from XPS of Li_*x*_S_*y*_ and ToF-SIMS of Na_*x*_S_*y*_ imply that a compositional gradient was formed over the film depth, with a higher Na-to-S ratio close to the metal. This is expected from the gradient of the chemical potential of Na and is analogous to the metal-to-oxygen ratio in oxides (*e.g.,* in oxides on Fe).^55,56^

**Figure 3:**
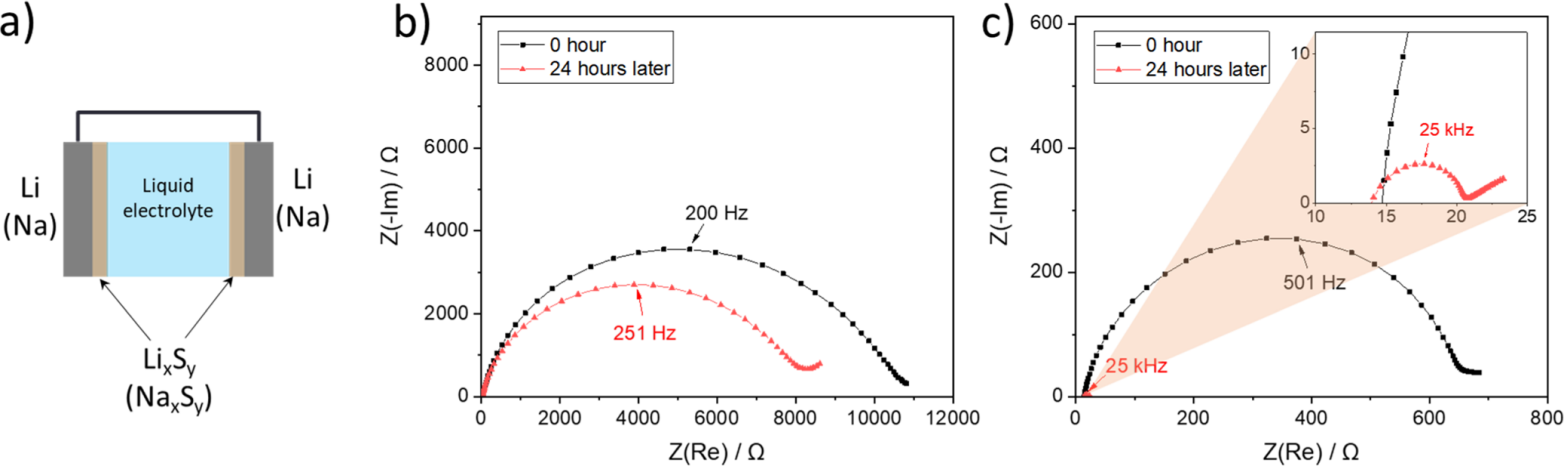
(a) Schematic diagram of the electrochemical measurement cell configuration, and EIS results of (b) Li|Li_*x*_S_*y*_|liquid electrolyte|Li_*x*_S_*y*_|Li and (c) Na|Na_*x*_S_*y*_|liquid electrolyte|Na_*x*_S_*y*_|Na. The liquid electrolyte used is 1 M LiCF_3_SO_3_(NCF_3_SO_3_) in triglyme. The impedance was measured right after cell assembly and after 24 h of aging under OCP. The inset in (c) shows the small semicircle observed at medium frequencies after 24 h of OCP aging.

In order to examine the role of the synthesized films (Li_*x*_S_*y*_ and Na_*x*_S_*y*_) on Li and Na as protective layers, they were assembled into symmetric cells (Figure 3a) with glyme-based liquid electrolytes, one of the most commonly used electrolyte classes for Li–S and Na–S batteries.^57,58^ The combination of a triflate salt and triglyme was chosen since a small interfacial resistance, *R*_interface_, has been reported in this case, especially for Na.^59,60^ In both cases, a decrease of the interfacial resistance was observed for open-circuit potential (OCP) aging of cells (Figure 3b,c) together with a change to yellow or orange-like color of the electrolyte solution (when performed in a beaker cell), which most likely indicates the dissolution of the polysulfide species.^61,62^ This behavior (*i.e., R*_interface_ decrease with time) was also reported in the literature when Li_2_S was synthesized on Li and tested in battery cells.^34^ In the case of Na symmetric cells with Na_*x*_S_*y*_, the interfacial resistance becomes as small as ∼5 Ω after 24 h. This might indicate that the molar proportion of sodium polysulfide is higher in Na_*x*_S_*y*_ than in Li_*x*_S_*y*_, or this could simply be due to the smaller amount of Na_*x*_S_*y*_ compared to Li_*x*_S_*y*_ (thinner layer). Another realistic picture is that the electrolyte penetrates through the pores (or cracks) in the surface films, reacting with fresh Li/Na, resulting in the formation of another type of SEI, and finally leading to a mechanical detachment of the already existing artificial SEIs (Li_*x*_S_*y*_ and Na_*x*_S_*y*_). If this is true, the more rapid decrease of interfacial resistance in the case of the Na symmetric cell with Na_*x*_S_*y*_ (Figure 3c) compared to the Li symmetric cell with Li_*x*_S_*y*_ (Figure 3b) indicates that the Na_*x*_S_*y*_ film is much more porous than Li_*x*_S_*y*_. This is also expected, based on the fact that Na_*x*_S_*y*_ appears to be rougher than Li_*x*_S_*y*_, as observed by FIB-SEM (Figure 2a,d). A more detailed investigation, such as quantitative analyses for the determination of the amount of different sulfide phases in Li_*x*_S_*y*_ and Na_*x*_S_*y*_ films and cryo-TEM to observe their porosity, would assist in the clarification of the origin of the different electrochemical behaviors of Li_*x*_S_*y*_ and Na_*x*_S_*y*_. This is, however, beyond the scope of the here-presented work. Additionally, highly concentrated electrolytes [*i.e.,* 5 M lithium bis(trifluoromethanesulfonyl)imide (LiTFSI) in dioxolane/dimethylether (DOL/DME) (1:1 vol) and 5 M sodium bis(fluorosulfonyl)imide (NaFSI) in DME], which were reported to show excellent electrochemical performance in batteries,^63,64^ were employed in Li and Na symmetric cells with and without artificial SEIs (Figure S9, Supporting Information). In all cases, increase of *R*_interface_ was observed, especially for the Na case, which implies that the highly concentrated electrolyte is still reactive, rendering a fair comparison between Li_*x*_S_*y*_ and Na_*x*_S_*y*_ difficult.

**Figure 4:**
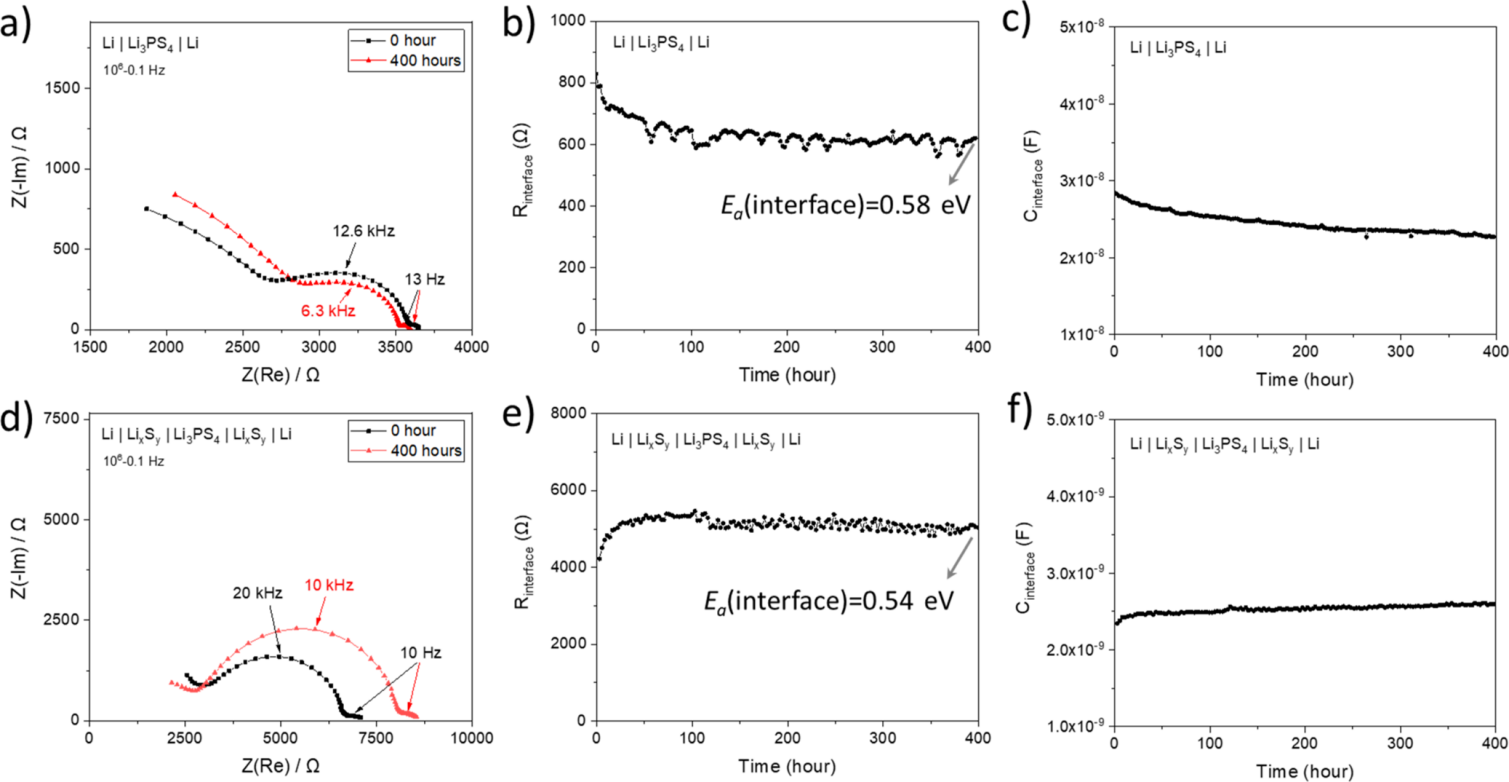
Investigation of the time-dependent growth of interfacial contributions with EIS under open-circuit conditions in Li|Li_3_PS_4_|Li and Li|Li_*x*_S_*y*_|Li_3_PS_4_|Li_*x*_S_*y*_|Li. (a,d) Nyquist plots showing all contributions. (b,e) Change in interface resistances (*R*_interface_) over time. (c,f) Change in capacitances corresponding to the interface (*C*_interface_) over time. More details on the *E*_a_(interface) values can be found in the Supporting Information (Figure S8).

**Figure 5:**
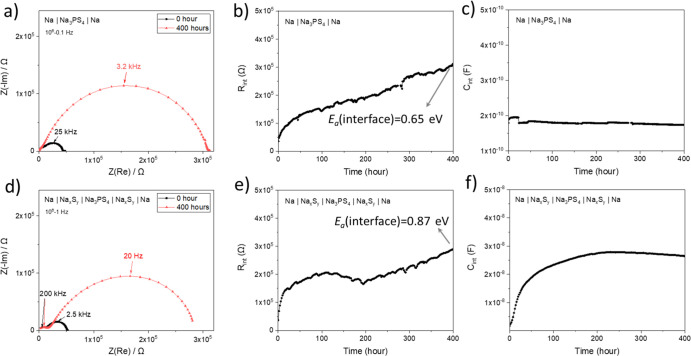
Investigation of the time-dependent growth of interfacial contributions with impedance spectroscopy under open-circuit conditions in Na|Na_3_PS_4_|Na and Na|Na_*x*_S_*y*_|Na_3_PS_4_|Na_*x*_S_*y*_|Na. (a,d) Nyquist plots showing all contributions. (b,e) Change in interface resistances (*R*_interface_) over time. (c,f) Change in capacitances corresponding to the interface (*C*_interface_) over time. More details on the *E*_a_(interface) values can be found in the Supporting Information (Figure S8).

We can circumvent the issue of the dissolution of the polysulfide species by using solid-state cells. Sulfide-based solid electrolytes β-Li_3_PS_4_ and cubic Na_3_PS_4_ were synthesized *via* a solvent-mediated synthetic route and a mechanical ball milling procedure, respectively. XRD of the synthesized solid electrolytes identifies that the crystal phases (β-Li_3_PS_4_ and cubic Na_3_PS_4_) are formed (Figure S4a,c Supporting Information). We measured the ionic conductivities of β-Li_3_PS_4_ and cubic Na_3_PS_4_ to be 1.47 × 10^–4^ and 1.55 × 10^–6^ S cm^–1^ (see Figure S4b,d, Supporting Information), by EIS with blocking Ru electrodes, respectively. Subsequently, symmetric cells consisting of Li(Na) electrodes with Li_*x*_S_*y*_(Na_*x*_S_*y*_) artificial SEIs and Li_3_PS_4_(Na_3_PS_4_) were assembled. We compare the EIS behavior of these cells with those of the bare Li/Na symmetric cells (Li|Li_3_PS_4_|Li and Li|Li_*x*_S_*y*_|Li_3_PS_4_|Li_*x*_S_*y*_|Li in Figure 4 and the analogous system with Na in Figure 5). In the Li case, three semicircles (Figure 4a,d) were observed, indicating at least three different charge transport processes appearing at different relaxation times (see Figure S5a, Supporting Information, where the equivalent circuit model used for fitting the impedance spectra is suggested). Ionic transport through the bulk solid electrolyte appears at the highest frequency (>20 kHz) as confirmed from the measurement with blocking electrodes (Figure S4b, Supporting Information). The semicircle at medium frequencies (maximum frequency between 6 and 20 kHz) corresponds to the interfaces between Li and Li_3_PS_4_ as they continuously change over time. We believe that the change in the interfacial resistance (*R*_interface_) over time in the cell with bare Li (Figure 4b) is most likely due to the continuous formation of SEIs by a chemical reaction between Li and Li_3_PS_4_ according to^28^
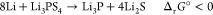
The origin of the smallest semicircle appearing at low frequency (<10 Hz) is not clear, but we suspect it stems from the imperfect solid contact or the charge transfer at the interface. Note that only the interfacial resistance changes over time, while the other two resistances (bulk conduction through the solid electrolyte, which appears at higher frequency, and contact resistance/charge transfer at lower frequency) stay constant over time (more details in Figure S6, Supporting Information).

The observed changes in the values of interfacial resistances (*R*_interface_) over time starkly vary in the two different systems. The *R*_interface_ value decreases in the case of a symmetric cell with bare Li (Figure 4b), which is counter-intuitive considering the fact that predominantly resistive phases (thermodynamically expected to produce more Li_2_S than Li_3_P with a molar ratio of 4:1, σ(Li_2_S) ∼ 10^–11^ S cm^–1^^30^ and σ(Li_3_P) ∼ 10^–4^ S cm^–1^^65^) are formed. This may be attributable to the creep of Li through the SEI due to the external pressure built up in the coin cell.^24^ Another possibility is the partial electronic conductivity of Li_3_P. If this is a considerable value, Li_3_P would allow for significant chemical diffusion through the SEI, resulting in further chemical reactions. Therefore, the value of *R*_interface_ will decrease if the SEI morphologically rearranges in time in such a way that the contact between the Li_3_P phases improves or Li_3_P becomes connected in parallel to the other existing phases. The symmetric cell with the artificial Li_*x*_S_*y*_ SEI shows opposite behavior compared to the one with bare Li, where the value of *R*_interface_ increases with time (first 100 h, Figure 4e). This indicates a continuous formation of resistive phases, which may be Li_2_S and/or LiOH produced by a chemical reaction between the protruded Li and polysulfides or a reaction between the residual H_2_O (on the surface of Li_*x*_S_*y*_) and Li_3_PS_4_. Another possibility is crack formation in the artificial Li_*x*_S_*y*_ SEI during the electrochemical cell assembly; this would most likely influence the *R*_interface_ values of the initial measurement. Note that in both cases, the activation energy of conduction (*E*_a_) is measured to be 0.54–0.58 eV, suggesting that both Li_3_P (*E*_a_ = 0.44 eV^65^) and Li_2_S (*E*_a_ = 0.90 eV^30^) contribute to the electrical conduction. The calculated capacitance of the order of 10^–9^ F in the case of the symmetric cell with Li_*x*_S_*y*_ (Figure 4e) is in good agreement with the expected capacitance if the thickness of Li_*x*_S_*y*_ measured by FIB-SEM (Figure 2a) is taken into account together with the dielectric constant of Li_2_ S (ε = 11).^30^ Symmetric cells with bare Li show capacitance values one order of magnitude higher than the one with Li_*x*_S_*y*_, indicating a thinner SEI or considerable contributions from the grain boundaries. Based on the previous literature dealing with the Li|Li_3_PS_4_ interface, in which almost no degradation is observed by FIB-SEM,^66^ it is more likely that a very thin SEI forms in the case of the symmetric cells with bare Li.

Figure 5 shows the impedance evolution over time in the Na symmetric cells with and without the artificial sulfide-based SEI (Na|Na_3_PS_4_|Na and Na|Na_*x*_S_*y*_|Na_3_PS_4_|Na_*x*_S_*y*_|Na). In both cases, two semicircles were observed (Figure 5a,d) in the impedance spectra, which were fitted with an equivalent circuit model including two RC parallel circuits in series (Figure S5b, Supporting Information). The semicircle appearing at high frequency (>100 kHz) corresponds to the ionic transport through the bulk solid electrolyte, while the semicircle at low frequency (<100 kHz) represents the interface between Na and the solid electrolyte as it changes gradually over time (Figure S6, Supporting Information). The expected interface formation reaction is symmetric to the one of Li.^29^ The interfacial resistance (*R*_interface_) increases continuously for both the pristine Na symmetric cells and the ones with a Na_*x*_S_*y*_ layer (Figure 5b,e). There have been reports in the literature about the increase of *R*_interface_ in Na|Na_3_PS_4_.^24,67^ The underlying reason is suspected to be the partial electronic conductivity of Na_3_P affecting the interface evolution similarly as described for the Li case,^67−69^ but the defect chemistry of this compound has not been experimentally confirmed due to its explosiveness and the potential risk of releasing a toxic gas (PH_3_). Another possibility is that Na creeps through the pores of the artificial SEI, reacting with Na_3_PS_4_ instantly upon contact and producing another SEI. This issue is still under discussion, though, because it is generally believed that the metal creep induces a drop in *R*_interface_ in electrochemical cells due to the high electrical conductivity of metals.^24^ Regardless of the similarity in the *R*_interface_ growth behavior of Na symmetric cells with and without a Na_*x*_S_*y*_ layer, the values of the interfacial capacitance are rather different. *C*_interface_ in a symmetric bare Na (without the artificial SEI) cell is in the order of 10^–10^ F and its value stays more or less constant over time (Figure 5c), while *C*_interface_ in a symmetric Na cell with a Na_*x*_S_*y*_ layer increases over time and finally reaches values in the order of 10^–8^ F (Figure 5f). Since it is unlikely that the thickness of the SEI decreases over time, an increase of *C*_interface_ may be attributable to the additionally formed interphases within the pores of the Na_*x*_S_*y*_ layer, contributing to the capacitance in a parallel way. Additionally, the activation energy of interfacial conductivity (*E*_a_) in the Na symmetric cell with the Na_*x*_S_*y*_ layer (0.87 eV) reveals that the major conduction pathway has a higher activation energy value than in the cell without the Na_*x*_S_*y*_ layer (0.65 eV) and even higher than the one of both Na_3_P (0.13 eV)^68^ and Na_2_S (0.59 eV).^31^ This may be attributable to the fact that (i) sodium polysulfide species have an inherently higher activation energy compared to Na_2_S or (ii) the Na_*x*_S_*y*_ layer has a porous structure in which electrical conduction is difficult. Also, having different *E*_a_ in the two Na systems (*i.e.,* Na|Na_3_PS_4_|Na and Na|Na_*x*_S_*y*_|Na_3_PS_4_|Na_*x*_S_*y*_|Na) means that the degree of change in *R*_interface_ values with temperature varies in these systems and that *R*_interface_ in the Na symmetric cell with Na_*x*_S_*y*_ might be lower than in the one without Na_*x*_S_*y*_ at low temperature.

**Figure 6:**
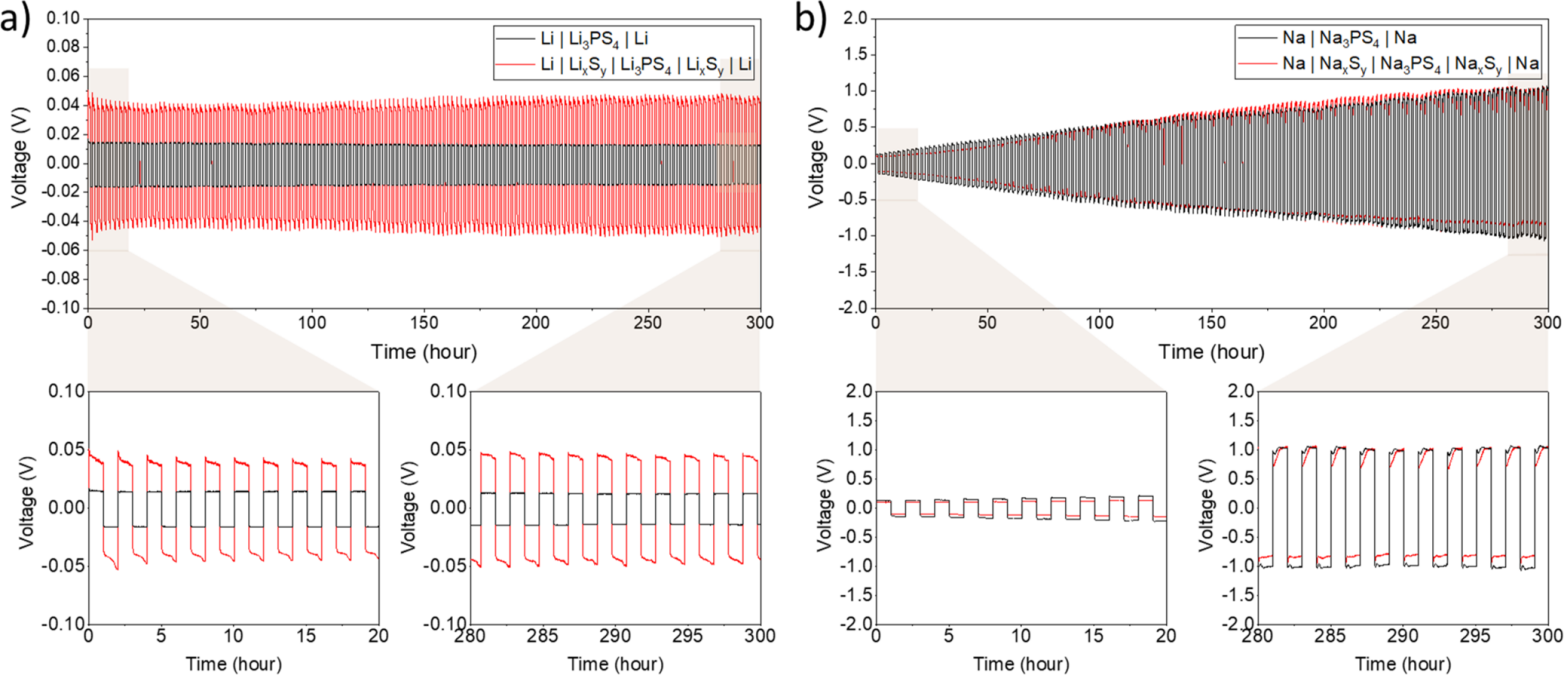
Stripping/plating behaviors of (a) symmetric Li cells with (red) and without (black) artificial sulfide-based SEI (Li_*x*_S_*y*_) and (b) symmetric Na cells with (red) and without (black) artificial sulfide-based SEIs (Na_*x*_S_*y*_). The applied current is 0.01 mA cm^–2^. Below each graph, the stripping/plating behavior of the first 20 h and the last 20 h are displayed.

Finally, galvanostatic stripping/plating of the four different systems (Li|Li_3_PS_4_|Li, Li|Li_*x*_S_*y*_|Li_3_PS_4_|Li_*x*_S_*y*_|Li, and the sodium variants) was performed (Figure 6) in order to determine their practical capability for battery application. For the Li case, the artificial SEI (*i.e.*, Li_*x*_S_*y*_) induces a considerably higher overpotential (Figure 6a, red) compared to the situation without Li_*x*_S_*y*_ (Figure 6a, black). Such an overpotential is especially large at the very first stage of stripping/plating (∼0.05 V) and gradually decreases afterward, implying that the nucleation energy barrier is higher compared to the case of bare Li. After nucleation started, the voltage of the cell with Li_*x*_S_*y*_ (red) is still higher than that of the cell with bare Li (black), implying that the artificial SEI layer (Li_*x*_S_*y*_) is more resistive than the interface formed by the contact between Li and Li_3_PS_4_. This higher overpotential in the Li_*x*_S_*y*_ case is consistent with the impedance results shown in Figure 4. A remarkable fact is that the Li|Li_3_PS_4_|Li and Li|Li_*x*_S_*y*_|Li_3_PS_4_|Li_*x*_S_*y*_|Li cells operate in a stable manner without a significant overpotential increase on galvanostatic cycling, implying that both the naturally formed SEI and the artificial SEI act as stable passivation layers in a chemo-mechanical sense. In the Na case, symmetric cells with and without the artificial SEI (Na_*x*_S_*y*_) both show similar cycling behavior (Figure 6b) in terms of overpotential values and the degree of overpotential increase on galvanostatic cycling, which is also in agreement with the resistance measured by EIS in Figure 5. In addition, the degree of overpotential increase is comparable under the OCP condition and under current application, implying that the applied current does not accelerate the growth of the SEI. The notable difference in Na cells compared to Li cells is that the overpotential increase was observed in both cases (*e.g.,* cells with and without Na_*x*_S_*y*_) as the stripping/plating progresses. The increase of overpotential on electroplating most likely indicates that Na^+^ diffusion is the rate-limiting factor rather than the Na nucleation,^70−72^ which might be attributed to the highly resistive SEI between Na and the electrolyte in both cases of the cell, that is, with and without Na_*x*_S_*y*_. Based on the galvanostatic stripping/plating results, it is concluded that the Na_*x*_S_*y*_ layer does not play a significantly different role in Na^+^ conduction compared to the SEI layer formed by the contact between Na and Na_3_PS_4_, highly likely due to the porosity of Na_*x*_S_*y*_.

## 4 Conclusions

Artificial sulfide-based SEIs (Li_*x*_S_*y*_ and Na_*x*_S_*y*_) were synthesized on Li and Na by a chemical reaction between Li/Na and S vapor. Chemical characterizations of these artificial SEIs suggest that they consist of polysulfides (Li_2_S_*y*_ and Na_2_S_*y*_, *y* > 1) and sulfides (Li_2_S and Na_2_S) with compositional gradients (Li/Na and S over the film depth), and a similar chemistry was observed in sputter-deposited lithium sulfide thin films in the literature.^53^ Given the fact that lithium polysulfide species are thermodynamically unstable as solid-state compounds,^73^ it is likely that various lithium polysulfide species exist as metastable phases only in the vicinity of the surface. The combination of morphology observation by FIB-SEM upon synthesis and OCP aging in symmetric cells with liquid organic electrolytes implies that Na_*x*_S_*y*_ is more porous compared to Li_*x*_S_*y*_, which might be due to the different *R*_PB_ of Li_2_S and Na_2_S/Na polysulfides [*R*_PB_(Li_2_S) = 1.06, *R*_PB_(Na_2_S) = 0.89, *R*_PB_(Na_2_S_2_) = 1.16, *R*_PB_(Na_2_S_4_) = 1.79, and *R*_PB_(Na_2_S_5_) = 2.17]. Note that this statement is only valid for the artificial SEI synthesized by the solid–vapor chemical reaction method. When the artificial SEIs are used in solid-state electrochemical cells, they do not provide advantages in terms of chemical stability of the interface or overpotential during stripping/plating. However, systematic comparison between Li and Na suggests that Na is much more reactive than Li against thiophosphate solid electrolytes, leading to the statement that Na requires an improved protection layer in terms of density and chemical stability. Also, we speculate that the reason why Na_*x*_S_*y*_ did not behave as a good protection layer in a solid-state electrochemical cell is most likely due to its nanoporosity, which could be proven by cryo-TEM in future work. We again stress the importance of examining the porosity of SEIs, not only for the ones formed by the contact between Li/Na and electrolytes but also for artificial SEIs in both liquid and solid electrolytes.^52^ Additionally, the effect of external pressure needs to be systematically investigated in the future in order to examine how Li/Na creep influences the interfacial resistance.

## Supporting Information Available

Cross-section images of Li_x_S_y_ on Li synthesized at various temperatures measured by FIB-SEM; cross-section images of Na_x_S_y_ on Na synthesized at various temperatures measured by FIB-SEM; comparison of R_PB_ of Li(Na) sulfides and polysulfides; energy-dispersive x-ray spectra of Li_x_S_y_ on Li and Na_x_S_y_ on Na; binding energies and full width at half-maximum of the S 2p_3/2_ peak for identification of different species by XPS; XRD patterns and EIS spectra of the synthesized β-Li_3_PS_4_ and cubic Na_3_PS_4_; equivalent circuit models for fitting the spectra of Li symmetric cells with Li_3_PS_4_ and Na symmetric cells with Na_3_PS_4_; evolution of the different resistance over time in the four systems; evolution of the different capacities in the four systems; Arrhenius plots and determination of the activation energy of electrical transport through the bulk SEI; and EIS results of symmetric Li and Na cells with highly concentrated electrolytes (PDF)
