# Supplementary material for: Influence of Porosity of Sulfide-Based Artificial Solid Electrolyte Interphases on Their Performance with Liquid and Solid Electrolytes in Li and Na Metal Batteries
Source: ACS Appl Mater Interfaces. 2022 Mar 31;14(14):16147–56. doi: 10.1021/acsami.1c23923 (PMC9011351; doi:10.1021/acsami.1c23923)
Supplement: Supplementary file 1 — am1c23923_si_001.pdf [file am1c23923_si_001.pdf]

Supporting Information

Influence of Porosity of Sulfide-Based Artificial Solid Electrolyte Interphases on their Performance with Liquid and Solid Electrolytes in Li(Na) Metal Batteries

Kyungmi Lim, Bernhard Fenk, Kathrin Küster, Tolga Acartürk, Jürgen Weiss, Ulrich Starke, Jelena Popovic*, Joachim Maier

Max Planck Institute for Solid State Research, 70569 Stuttgart, Germany

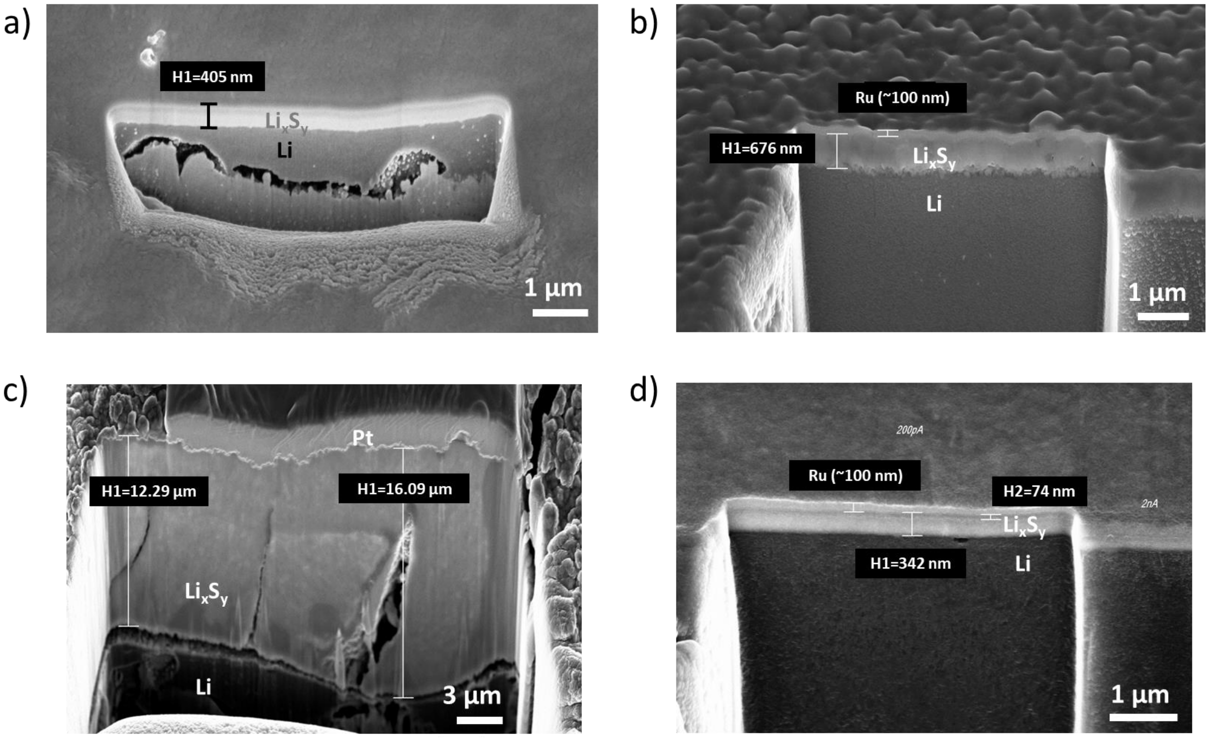

Figure S1. Cross-section images of Li_xS_y on Li synthesized at various temperature measured by focused-ion beam scanning electron microscopy (FIB-SEM). (a) $T_{Li}=T_S=60$ ˚C, (b) $T_{Li}=T_S=100$ ˚C, (c) $T_{Li}=T_S=120$ ˚C, (d) $T_{Li}=120$ ˚C, $T_S=25$ ˚C. The synthesis proceeded for 2 hours. In b), c), d), films were sputtered by Pt and Ru prior to the measurements, respectively. Note that cracks in (a) and (c) are formed during milling with focused ion beam.

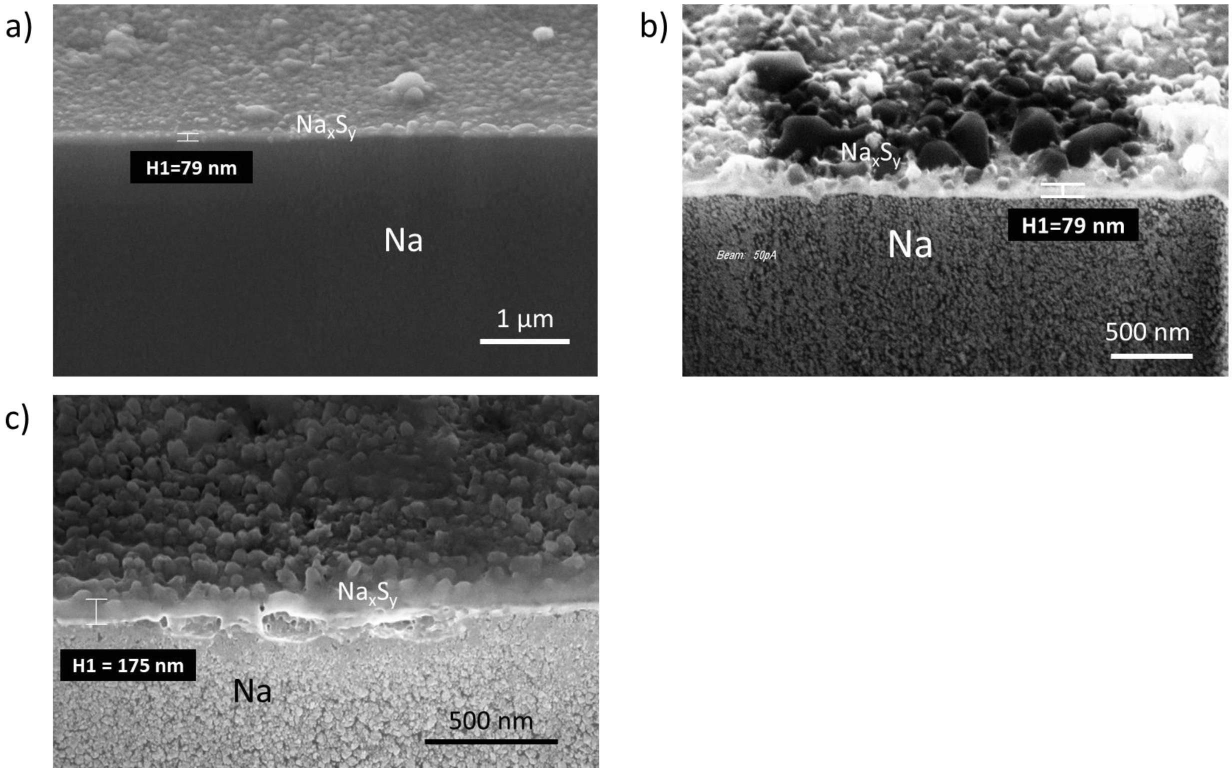

Figure S2. Cross-section images of Na_xS_y on Na synthesized at various temperature measured by FIB-SEM. (a) T_{Na}=60 ˚C, T_S=25 ˚C (b) T_{Na}=T_S=60 ˚C, (c) T_{Na}=T_S=70 ˚C. The synthesis proceeded for 2 hours.

Table S1. Comparison of Piling-Bedworth ratio (R_{PB}) of Li(Na) sulfides and polysulfides.

**Compound**	**R_{PB}**	**Compound**	**R_{PB}**
Li_2S	1.06	Na_2S	0.89
		Na_2S_2	1.16
		Na_2S_4	1.79
		Na_2S_5	2.17

Pilling-Bedworth ratio (R_{PB}) is the ratio of the molar volume of tLi(Na) sulfides/polysulfides to the molar volume of metallic Li/Na.

$$2Li\ (or\ 2Na) + yS \rightarrow Li_2S_y\ (or\ Na_2S_y)$$

R_{PB} was calculated from

$$R_{PB} = \frac{V_{Li_2S_y\ (Na_2S_y)}}{2 \times V_{Li(Na)}} \quad (1)$$

where $V_{\mathrm{Li_2S_y\ (Na_2S_y)}}$ is the molar volume of Li/Na sulfide/polysulfides, $V_{Li(Na)}$ is the molar volume of metallic Li (Na).

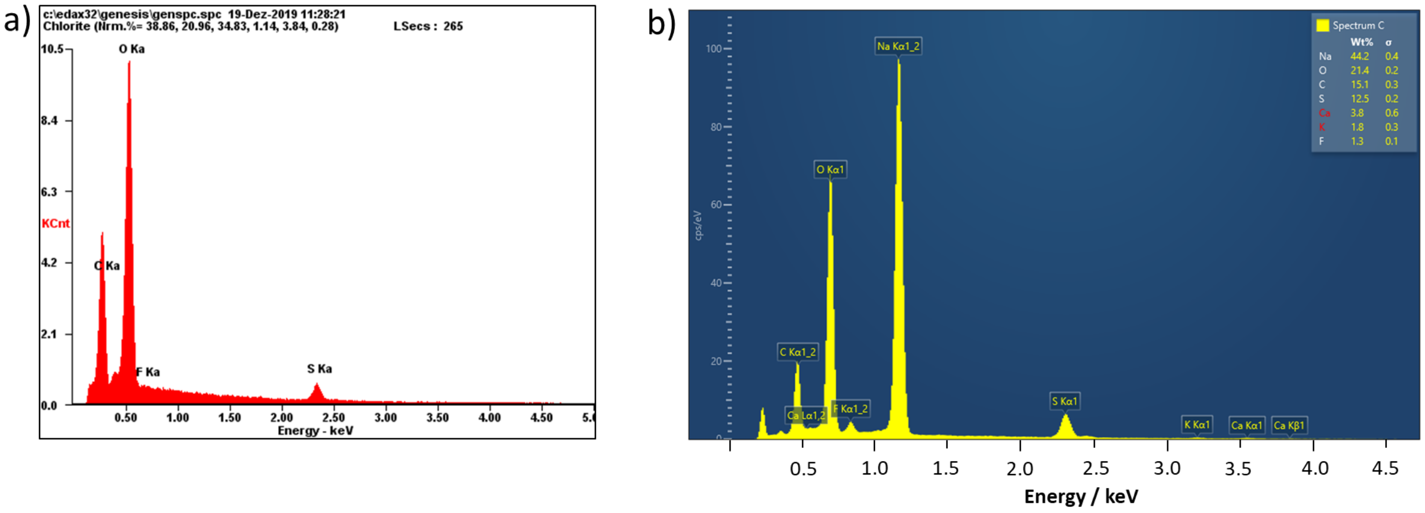

Figure S3. Energy dispersive X-ray spectra on (a) Li_xS_y on Li and (b) Na_xS_y on Na. C, O, F, Ca and K are common contaminants.

Table S2. Binding energies and full width at half maximum of the S $2p_{3/2}$ peak for identification of different sulfide species by XPS.

Species	Binding energy (eV)	FWHM (eV)	Reference
Li_2S (S^{2-})	162.1	0.86	[1]
Li_2S_2 (S^{-})	163.5	1.31	[2]
SO_3^{2-} (S^{4+})	167.6	0.80	[3]
SO_4^{2-} (S^{6+})	168.9	1.07	[4]

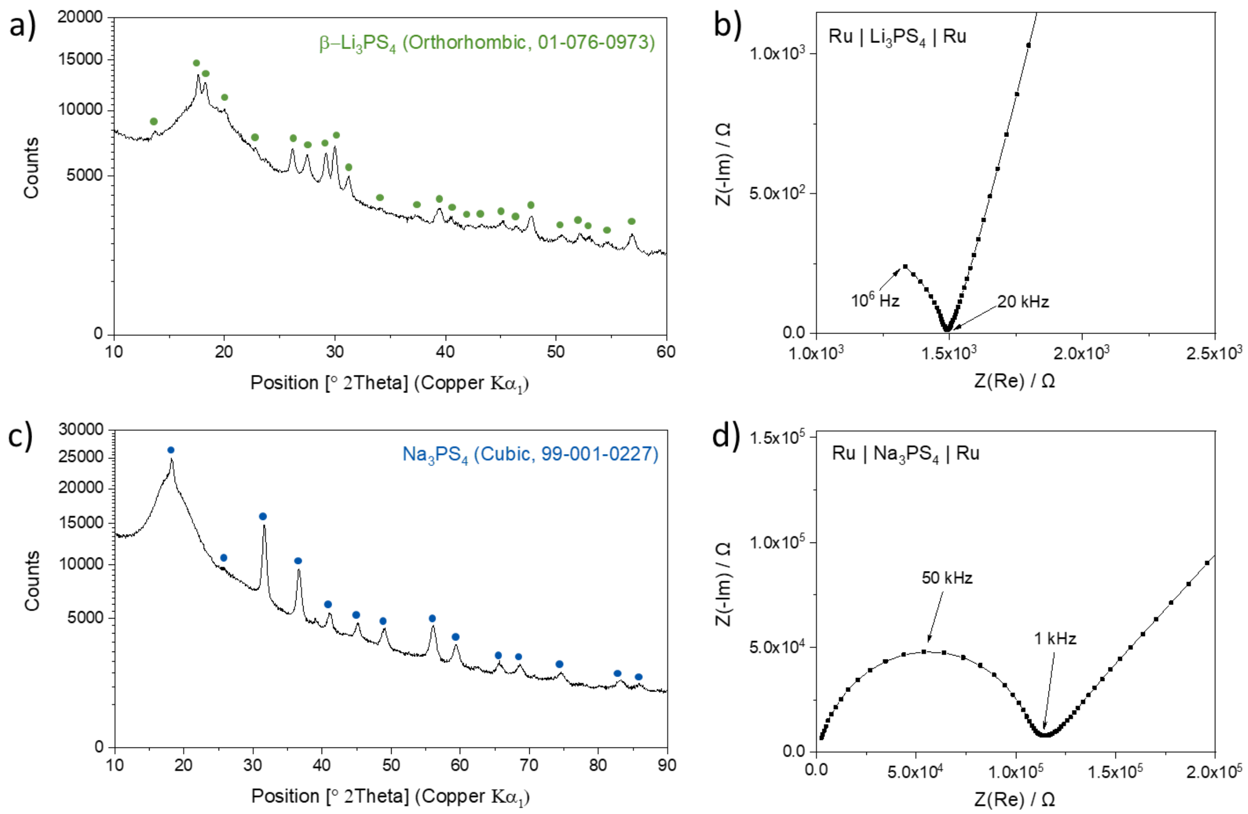

Figure S4. X-ray diffraction patterns and electrochemical impedance spectra (EIS) of synthesized β-Li_3PS_4 and cubic-Na_3PS_4. For measuring ionic conductivity of materials, Ru was sputter-deposited on both sides of the dense electrolyte pellets in a glovebox.

Ionic conductivities of Li_3PS_4 and Na_3PS_4 were calculated following:

$$\sigma = \frac{L}{RA} \quad (2)$$

where σ is the ionic conductivity of the sample, L is the thickness of the pellet, R is the bulk electrolyte resistance measured by EIS and A is the area of a flat surface of the pellet.

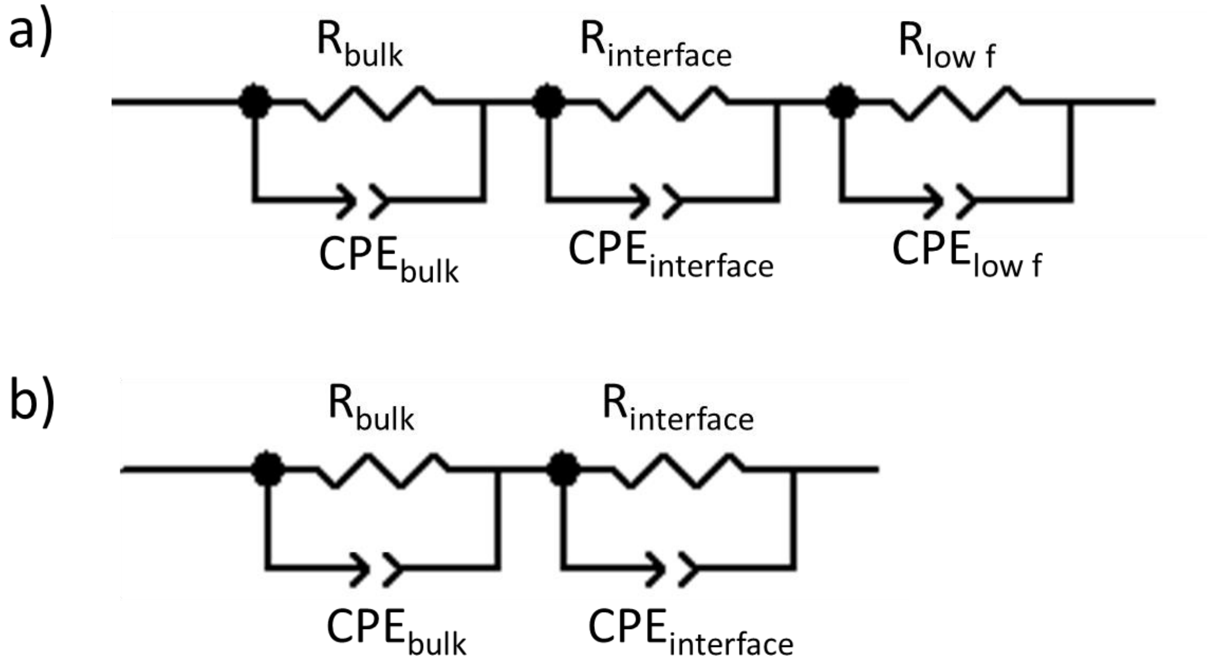

Figure S5. Equivalent circuit models for fitting spectra of (a) Li symmetric cells with Li_3PS_4 and (b) Na symmetric cells with Na_3PS_4. CPE: constant phase element. R_{bulk} and CPE_{bulk} correspond to bulk solid electrolyte appearing at high frequency (>20 kHz), and $R_{interface}$ and $CPE_{interface}$ correspond to interface (SEI). $R_{low\ f}$ and $CPE_{low\ f}$ are resistance and constant phase element appearing at low frequency (< 10 Hz) in Li symmetric cells with Li_3PS_4.

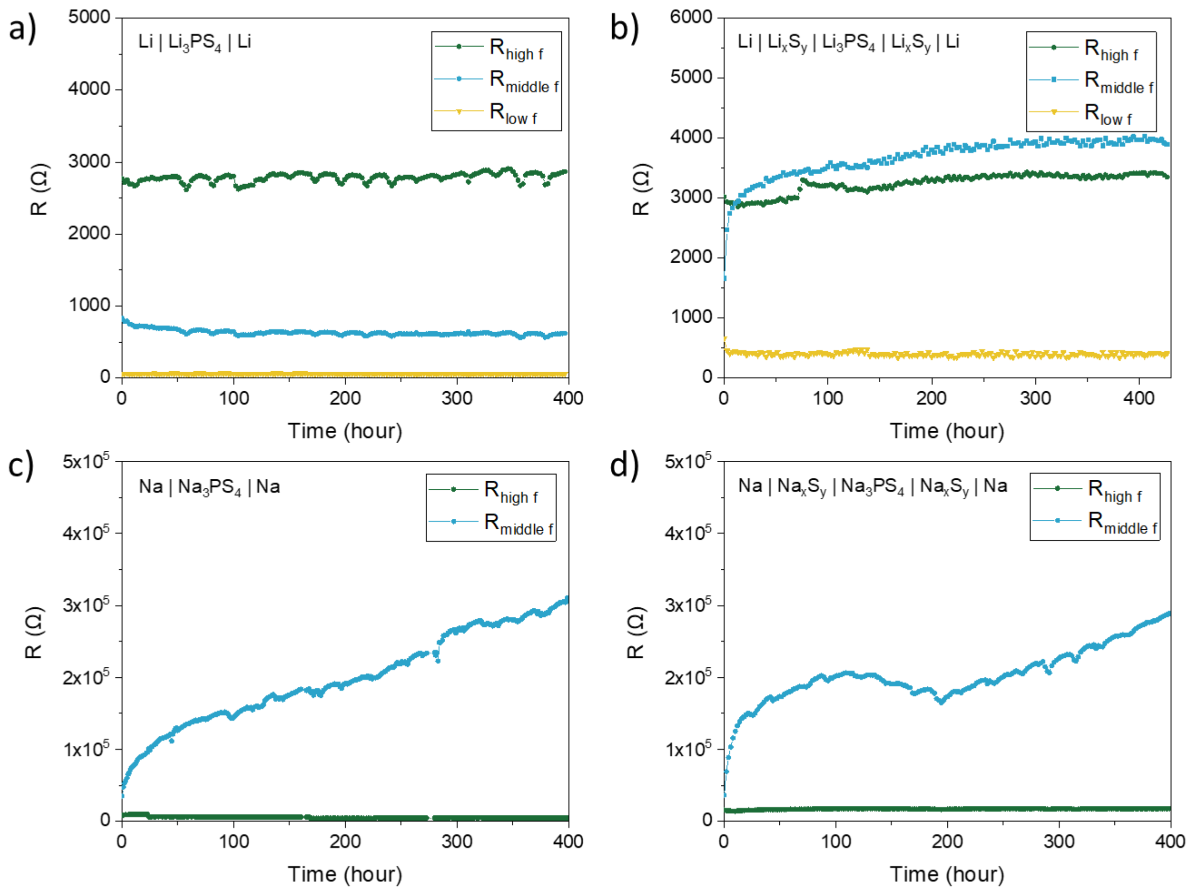

Figure S6. Evolution of the different resistances (semicircles appearing at high, middle and low frequencies) over time in four different systems. (a) $Li|Li_3PS_4|Li$, (b) $Li|Li_xS_y|Li_3PS_4|Li_xS_y|Li$, (c) $Na|Na_3PS_4|Na$, (d) $Na|Na_xS_y|Na_3PS_4|Na_xS_y|Na$

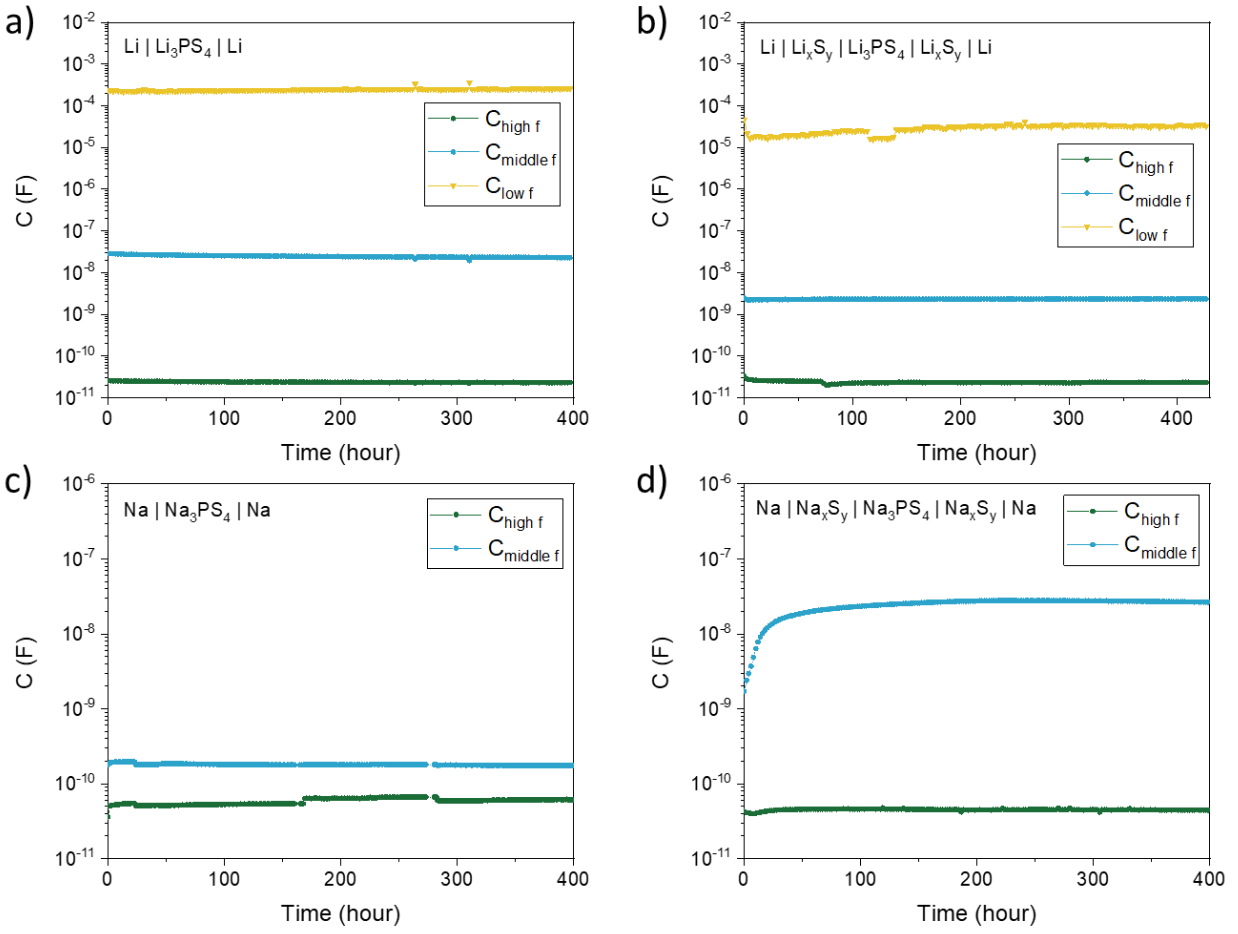

Figure S7. Evolution of the different capacitances (semicircles appearing at high, middle and low frequencies) over time in four different systems. (a) $Li|Li_3PS_4|Li$, (b) $Li|Li_xS_y|Li_3PS_4|Li_xS_y|Li$, (c) $Na|Na_3PS_4|Na$, (d) $Na|Na_xS_y|Na_3PS_4|Na_xS_y|Na$

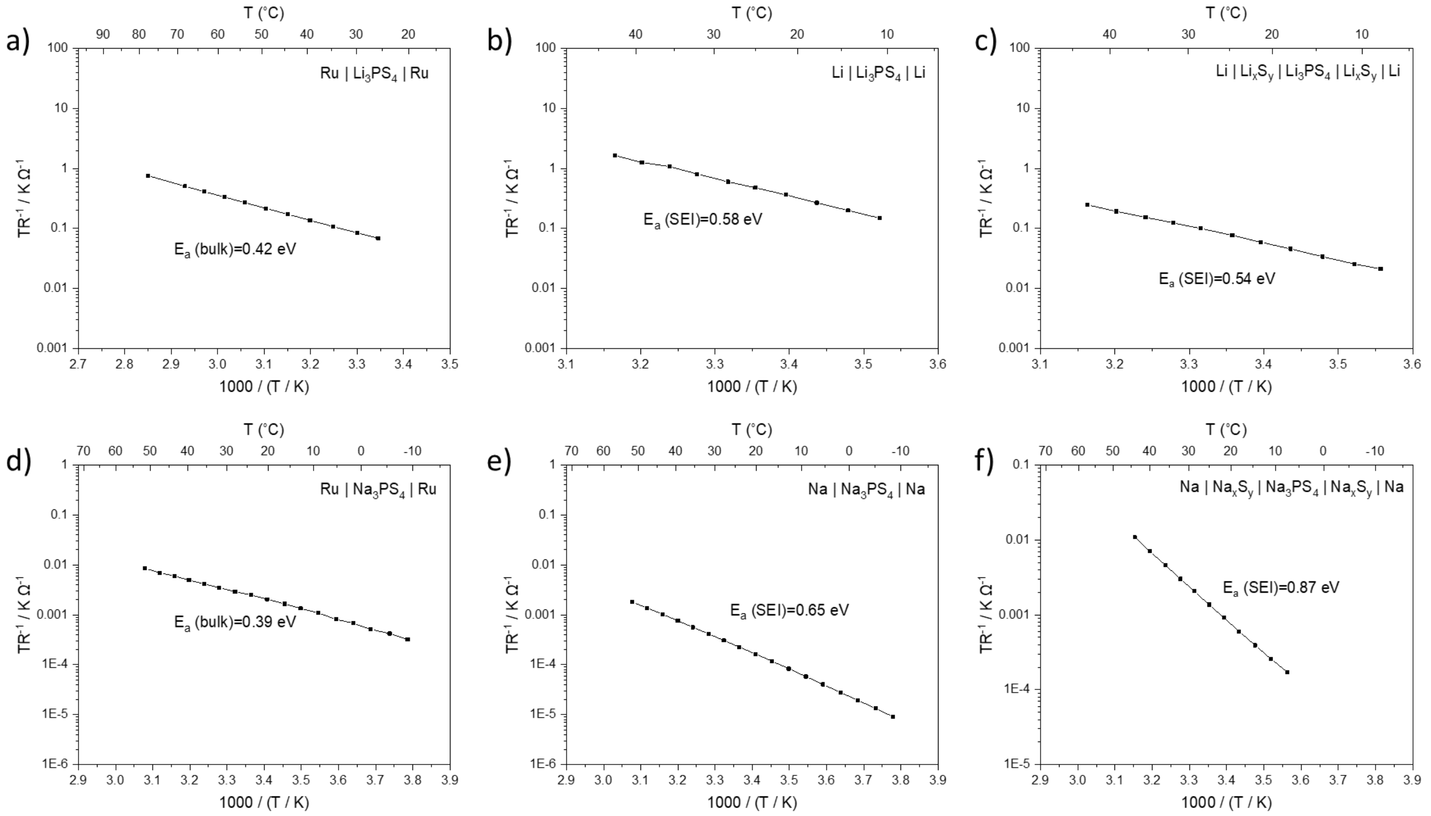

Figure S8. Arrhenius plots and determination of the activation energy of electrical transport through bulk solid electrolyte and interface (SEI).

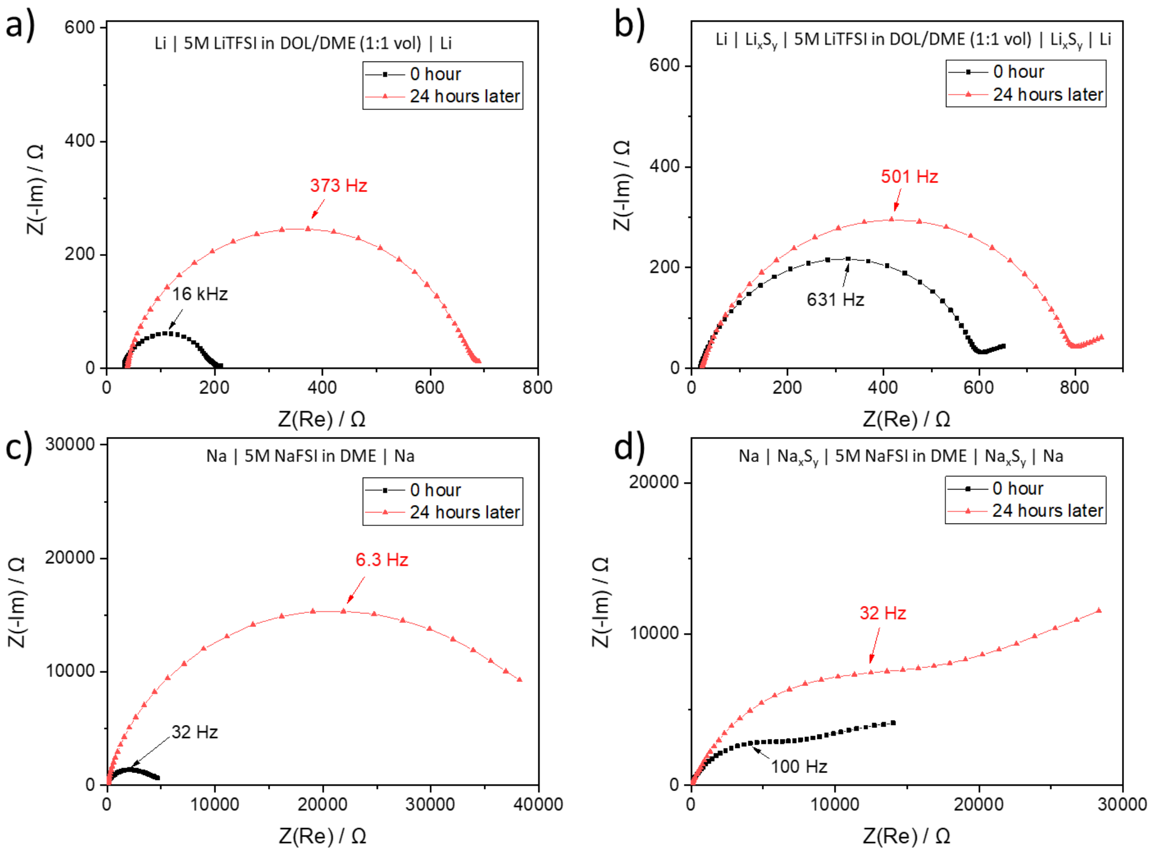

Figure S9. EIS results of symmetric (a,b) Li and (c, d) Na cells with highly-concentrated electrolytes. LiTFSI: Lithium bis(trifluoromethanesulfonyl)imide, DOL: 1,2-dioxolane, DME: dimethoxyethane, NaFSI: Sodium bis(fluorosulfonyl)imide. Electrochemical cells were stored under OCP condition and Li/Na cells were compared with and without Li_xS_y/Na_xS_y layers.

References

[1] K. M. Abraham et al., J. Electrochem. Soc. 133(7), 1307 (1986)

[2] M. Fantauzzi et al., RSC Adv. 5 75953 (2015)

[3] B. J. Lindberg et al., Physica Scripta 1 286 (1970)

[4] S. Contarini et al., J. Electron Spectrosc. Relat. Phenom. 35(2) 191 (1985)
